# Predicting climate-driven shift of the East Mediterranean endemic *Cynara cornigera* Lindl

**DOI:** 10.3389/fpls.2025.1461639

**Published:** 2025-02-20

**Authors:** Heba Bedair, Yehia Hazzazi, Asmaa Abo Hatab, Marwa Waseem A. Halmy, Mohammed A. Dakhil, Mubaraka S. Alghariani, Mari Sumayli, A. El-Shabasy, Mohamed M. El-Khalafy

**Affiliations:** ^1^ Botany Department, Faculty of Science, Tanta University, Tanta, Egypt; ^2^ United States Department of Agriculture (USDA) Forest Service, International Institute of Tropical Forestry, San Juan, Puerto Rico; ^3^ Department of Biology, College of Science, Jazan University, Jazan, Saudi Arabia; ^4^ Botany and Microbiology Department, Faculty of Science, Kafrelsheikh University, Kafrelsheikh, Egypt; ^5^ Department of Environmental Sciences, Faculty of Science, Alexandria University, Alexandria, Egypt; ^6^ School of Ecology and Environment, Northwestern Polytechnical University, Xi’an, China; ^7^ Botany and Microbiology Department, Faculty of Science, Helwan University, Cairo, Egypt; ^8^ Libyan Authority for Scientific Research, National Project for Disaster and Crisis Management in Libyan State, Tripoli, Libya; ^9^ Department of Geography and Geographic Information System (GIS), Faculty of Arts and Languages, University of Tripoli, Tripoli, Libya

**Keywords:** species distribution models, anthropogenic effects, conservation of natural resources, Mediterranean basin, sustainability

## Abstract

**Introduction:**

Climate change poses significant challenges to the distribution of endemics in the Mediterranean region. Assessing the impact of climate change on the distribution patterns of Mediterranean endemics is of critical importance for understanding the dynamics of these terrestrial ecosystems under the uncertainty of future changes. The population size of the *Cynara cornigera* has declined significantly over the previous century across its geographical region. This decline is linked to how ongoing climate change is affecting natural resources like water and the capacity of foraging sites. In fact, it is distributed in 3 fragmented locations in Egypt (Wadi Hashem (5 individuals), Wadi Um Rakham (20 individuals), Burg El-Arab (4 individuals)).

**Methods:**

In this study, we examined *C. cornigera*’s response to predicted climate change over the next few decades (2020-2040 and 2061-2080) using species distribution models (SDMs). Our analysis involved inclusion of bioclimatic variables, in the SDM modeling process that incorporated five algorithms: generalized linear model (GLM), Random Forest (RF), Boosted Regression Trees (BRT), Support Vector Machines (SVM), and Generalized Additive Model (GAM).

**Results and discussion:**

The ensemble model obtained high accuracy and performance model outcomes with a mean AUC of 0.95 and TSS of 0.85 for the overall model. Notably, RF and GLM algorithms outperformed the other algorithms, underscoring their efficacy in predicting the distribution of endemics in the Mediterranean region. Analysis of the relative importance of bioclimatic variables revealed Precipitation of wettest month (Bio13) (88.3%), Precipitation of warmest quarter (Bio18) (30%), and Precipitation of driest month (Bio14) (22%) as the primary drivers shaping the potential distribution of *C. cornigera*. The findings revealed spatial variations in habitat suitability, with the highest potential distribution observed in Egypt, (especially the Arishian sub sector), Palestine, Morocco, Northern Cyprus, and different islands in the Sea of Crete. Furthermore, our models predicted that the distribution range of *C. cornigera* would drop by more than 25% during the next few decades. Surprisingly, the future potential distribution area of *C. cornigera* (SSP 126 scenario) for 2061 and 2080 showed that there is increase in the suitable habitats area. It showed high habitat suitability along the Mediterranean coastal strip of Spain, Sardinia, Morocco, Algeria, Tunisia, Libya, Egypt, (especially the Arishian sub sector), Palestine, Lebanon, Northern Cyprus, and different Aegean islands.

## Introduction

Climate change represents a fundamental force reshaping plant species dynamic, impacting ecosystems and biodiversity worldwide (Weiskopf et al., 2020). The Mediterranean region, characterized by its unique climatic and ecological conditions, harbors a rich diversity of endemic plant species (Rundel et al., 2016; Bedair et al., 2022, 2023). Climate plays a pivotal role in shaping the dynamics of these Mediterranean endemics, exerting both direct and indirect influences on their distribution, phenology, and overall ecological interactions (Lead, 2020; Tuel and Eltahir, 2020). Rising temperatures, altered precipitation patterns, and changing climatic extremes associated with climate change pose significant challenges to the delicate balance of these species within their native ecosystems (Malhi et al., 2020). These shifts can affect crucial ecological processes such as germination, flowering, and fruiting, leading to changes in phenological events. Additionally, alterations in water availability and temperature regimes may impact the suitable habitats and geographic ranges of these endemics, potentially threatening their persistence (Parmesan and Hanley, 2015; Peñuelas et al., 2018). On the other hand, an increase in water availability and temperature might help plants to increase their geographical range. Results by showed that the suitable range of distribution for high-value medicinal plants would increase and concentrate in mountainous areas of central Nepal (Kunwar et al., 2023). As global temperatures rise and precipitation patterns undergo transformation, plant species must adapt or migrate to survive (Thornton et al., 2014).

In addition, Water shortage, a long-standing and widespread issue in many Mediterranean countries, is expected to worsen in the next decades as a result of anticipated climate change. Furthermore, agricultural practices designed to maximize yields have resulted in increased water use for irrigation. Continued intensification of agricultural methods will have negative repercussions for water supplies, biodiversity, and landscape function. This is associated with increased disruptions to environmental integrity, overexploitation of ecosystem services, and an increasing inclination toward desertification in certain rim countries (Cramer et al., 2018).

Based on the most recent temperature record, the mean annual temperature for the entire basin is approximately 0.4°C higher than the global average and 1.5°C above late 19th-century levels, with notable inter-annual variability (Chart 1). Over the same time period, significant sub-regional manifestations of the warming show local increases in mean annual temperatures ranging from 1.5 to 4°C. The overall change in climatic conditions has resulted in more frequent and more intense heat waves with midday temperatures continuously above 30 to 40°C and increasing periods of drought (Lange, 2020).

There are about 13.000 plant species endemic to the Mediterranean region (Thompson et al., 2005). The Mediterranean basin hosts about 12 endemic vascular plants per 100 km^2^ and has been recognized as a priority region for conservation in Europe, as well as one of the 34 major “biodiversity hotspots” on the planet (Mittermeier, 2005).

*C. cornigera* Lindl., is Top of Form among the East Mediterranean endemics that play a vital role in Mediterranean ecosystems, contributing to biodiversity and ecosystem functioning. The species is widely farmed for economic purposes all over the world (Elsayed et al., 2012). It also offers socioeconomic benefits, being used as food (Pieroni et al., 2022) and in traditional medicine for liver disorders (El Sohafy et al., 2013). The plant exhibits antioxidant, hepatoprotective, metal-chelating, and diuretic effects (Hegazy et al., 2015a). The species’ hepatoprotective and pharmaceutical activities, verified by multiple studies (El Sohafy et al., 2013, 2016; Hegazy et al., 2015b), are attributed to its rich content of bioactive compounds, including polyphenolics, flavonoids, sterols, terpenes, halogenated metabolites, and sesquiterpene lactones like cynaropicrin and cynarin.

*C. cornigera*, commonly known as the wild artichoke and spiny artichoke thistle, belongs to the family Asteraceae (IPNI, 2024; POWO, 2024) and is a key member of the Cynareae tribe in Egypt (Täckholm, 1974; Boulos, 2002). Its native range is restricted to the eastern Mediterranean, from the East Aegean Islands, Greece, Kriti, and Cyprus to Egypt and Libya (POWO, 2024). *C. cornigera* does not have a global IUCN Red List status currently (IUCN, 2025). It is not listed in the IUCN Red List of Threatened Species. However, it has been regionally assessed by Bedair (2023) to be Critically Endangered in Egypt as it is distributed in only 3 fragmented locations (Wadi Hashem, Wadi Um Rakham, Burg El-Arab). Depending on the phytogeographic regions that identified by Abdelaal et al. (2020), *C. cornigera* is distributed in Marioutico-Arishian sector in Egypt, with high density in the Marioutic sub-sector and less density in the Arishian subsector.

Species Distribution Models (SDMs) are crucial in ecological research for predicting shifts in species distribution under varying climate scenarios (Elith and Leathwick, 2009; Srivastava et al., 2019; Roy et al., 2022; Yang et al., 2023). As the global climate changes, SDMs help understand impacts on biodiversity and ecosystems (Shivanna, 2022; Bernatchez et al., 2023; Dakhil et al., 2024). They integrate biological and environmental data to predict future distribution changes (Elith and Leathwick, 2009; Roy et al., 2022), using variables like temperature, precipitation, and land cover (Elith and Leathwick, 2009; Miller, 2010; Bernatchez et al., 2023). Algorithms such as Maxent, Random Forest, and Boosted Regression Trees generate spatial predictions (Pradervand et al., 2014; Li and Wang, 2013; Zeng et al., 2022). SDMs predict distribution changes under different climate scenarios (Elith and Leathwick, 2009; Gould et al., 2014; Bernatchez et al., 2023), using climate models to simulate future conditions (Kattenberg et al., 1996). They identify critical factors for targeted conservation strategies (Zeng et al., 2022), informing conservation practices and management decisions to address potential range shifts and implement proactive measures (Shivanna, 2022). The SDM ‘ensemble’ approach, which combines predictions across different modelling methods, is believed to improve predictive performance, and is used in many recent SDM studies (Hao et al., 2020). As recent SDM studies have successfully improved predictive accuracy by combining models generated from several algorithms (Hao et al., 2020; Adhikari et al., 2022, 2023; Subedi et al., 2023), rather than a single algorithm in SDM, an ensemble modeling approach was used to develop habitat suitability models, combining multiple SDM algorithms. This study aimed to use SDM techniques to 1) clarify the complex interactions between *C. cornigera* and climate variables; 2) predict the potential distribution patterns of *C. cornigera*; and 3) evaluate future changes in its geographic range under various climate change scenarios. The results will offer insights into the species’ potential responses to climate change, guiding proactive measures for its conservation and sustainable management.

## Materials and methods

### Study area

**Figure 1:**
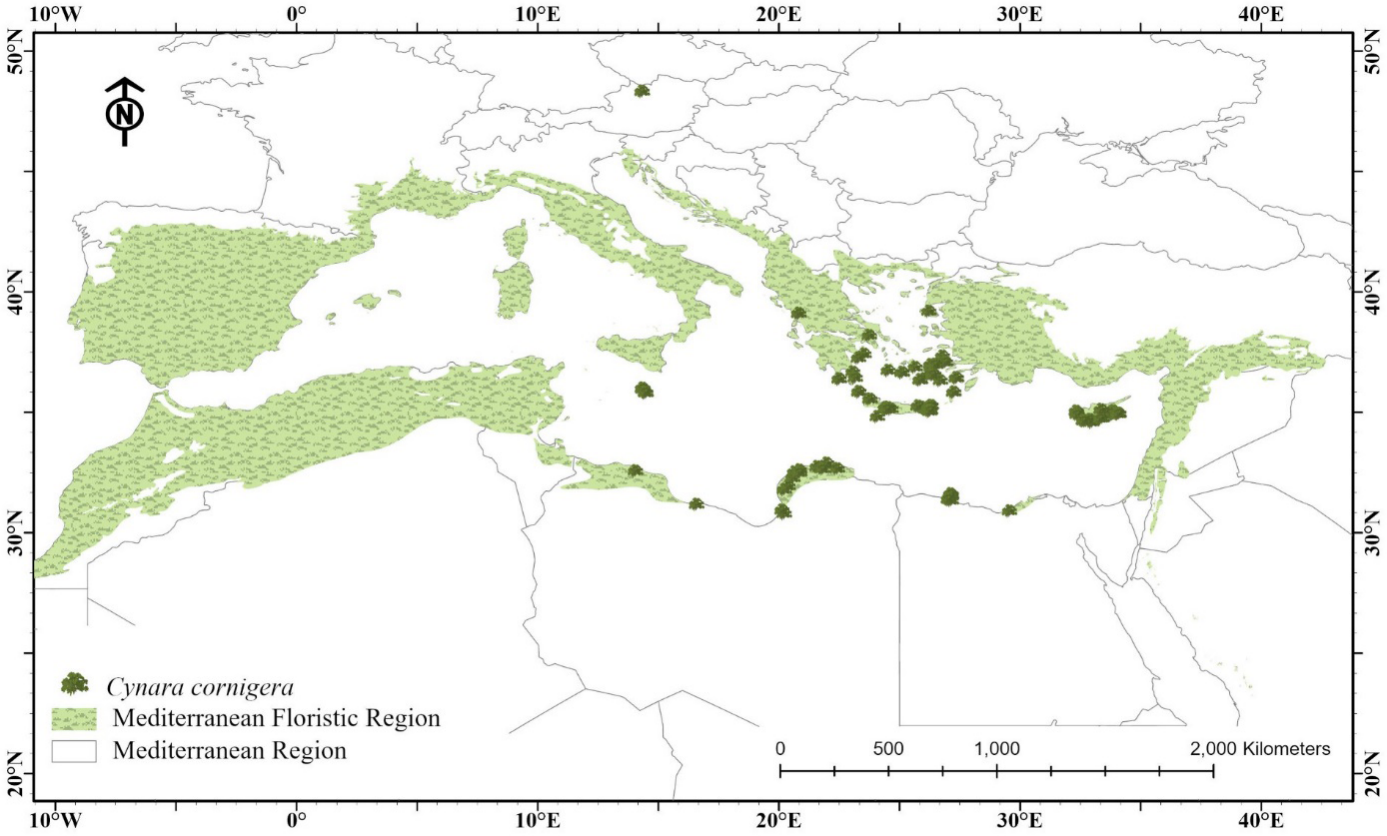
Map of the study area demonstrates the delineation of the Mediterranean floristic region (light green shading), and current occurrence locations of *C. cornigera* (dark green).

Delimitation of the Mediterranean region in this study was determined according to Bedair et al. (2023) after the system of Good (1974). It is important to remember that belts carrying species from adjacent contiguous regions divide regions from one another rather than lines. There is consensus in the Mediterranean region, in particular, regarding the identification of five vegetation belts (Vargas, 2020): 1- thermo-Mediterranean vegetation (coastal scrub and conifer-oak woodlands on inland hills); 2- meso-Mediterranean (sclerophyll woodland on plains and piedmont); 3- supra-Mediterranean (oak and pine woodlands up to the altitudinal timberline); 4- oro-Mediterranean (high-altitude bush communities); and 5- cryoro-Mediterranean (alpine communities of herbs). The boundaries of the main *Olea europaea* L. subsp. *europaea* cultivation area and the typical *Quercus ilex* L. Mediterranean forests coincide with the chorionomic boundary of the Mediterranean region (**Figure 1**). The climate is primarily Mediterranean in nature (temperate aridiestival), with significant thermal and ombric variability. With a preponderance of sclerophyllous and microphyllous woody plants, the vegetation exhibits adaptations to this environment. Lower trees with a lauroid form and a wider spread, such *Arbutus unedo*, *Laurus nobilis*, and *Viburnum tinus*, as well as lianas like *Smilax aspera*, appear in areas with more oceanic and humid conditions (Vynokurov et al., 2024).

**Figure 2:**
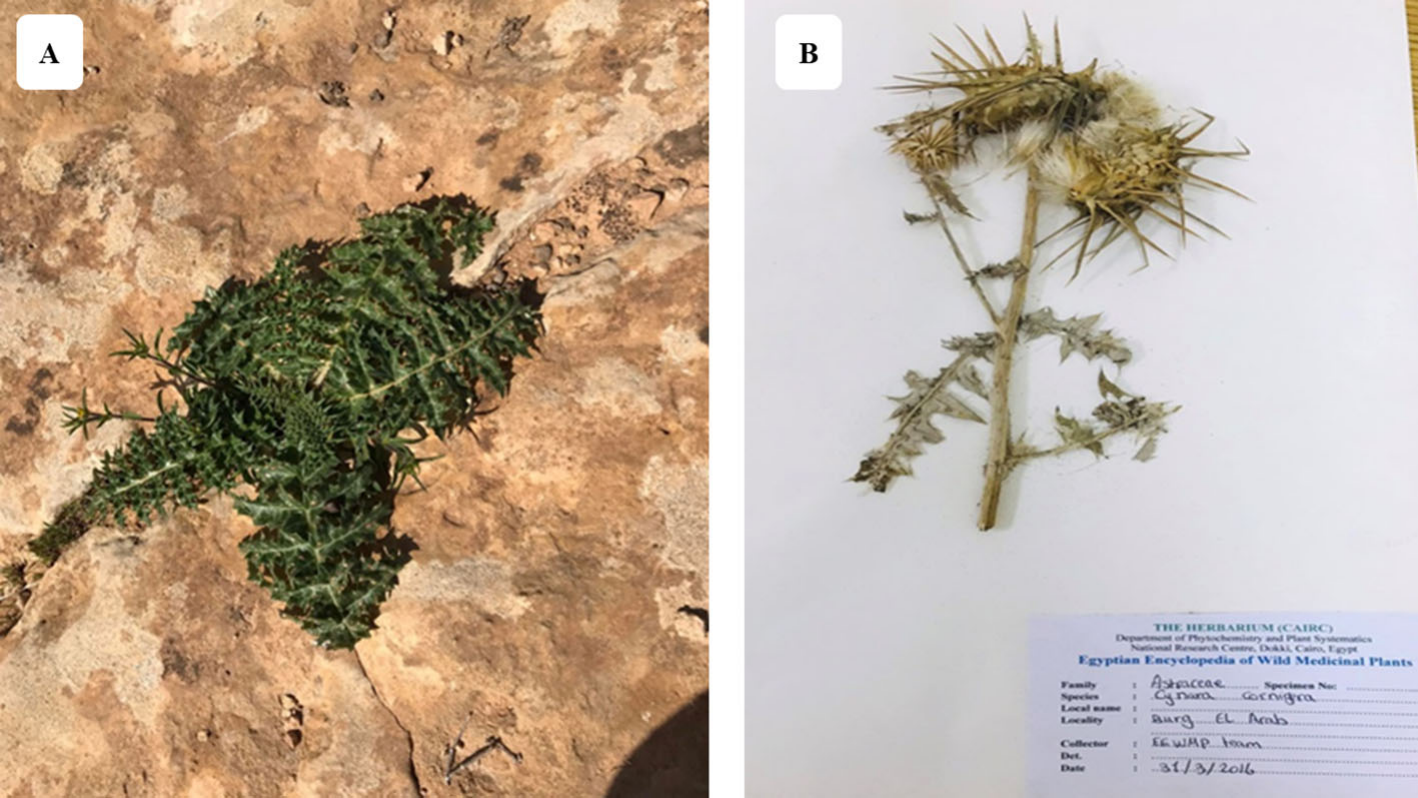
Photos of *C*. *cornigera*. **(A)** taken in Om El-Rakham, Mersa Matrouh, Egypt in March 2023, and **(B)** a sheet in herbarium specimen deposited in Notional Research Center, Egypt (Photos taken by H. Bedair).

*C. cornigera* (i. e. Kharshouf) is East Mediterranean endemic plant inhabiting numerous countries such as: Cyprus, East Aegean Is., Egypt, Greece, Kriti and Libya (Bedair et al., 2023) (**Figure 2**). In fact, it is a wild edible plant in many Mediterranean countries such as Egypt, Libya and Cyprus. It can be used as a vegetable or boiled with meat (Sumengen Ozdenefe et al., 2022). Indeed, it is regionally assessed as Critically Endangered by Shaltout et al. (2024) due to urbanization in the Mediterranean region, droughts, and over-collecting.

### Species occurrence data and cleaning

We obtained the distribution data (n= 449; 80 from field visits, 100 from herbarium sheets and the remaining from GBIF) of *Cynara cornigera* from 1. field visits, 2. the herbaria of Real Jardín Botánico de Madrid (MA), Tanta University (TANE), Alexandria University (ALEX), Cairo University (CAI), Assiut University (ASTU), Agricultural Research Center (CAIM), Desert Research Center (CAIH), National Research Centre (CAIRC) and Kafr Elsheikh University (KFSUH), 3. National Registry for Egyptian Herbaria (http://networks.asrt.sci.eg/Herbarias/Index), 4. iNaturalist (https://www.inaturalist.org), and 5. GBIF (*C. cornigera* Lindl. in GBIF Secretariat (2023). We cleaned the geographical data by removing duplicates and points outside the Mediterranean study area as well as outlier locations such as land use structures using the global land cover map in ArcGIS Pro (ESRI, USA).

### Bioclimatic predictors and multicollinearity

We downloaded the nineteen bioclimatic variables from the global database WorldClim 2.1 at the spatial resolution of 2.5 arcminutes (Fick and Hijmans, 2017). To predict the distribution of *C. cornigera* under climate change scenarios, we applied one of the commonly used global general circulation model (GCM): IPSL-CM6ALR for the near future (2021- 2040) and for the far future (2061–2080) for the recent two socioeconomic scenario pathways (low scenario: SSP126 and high scenario: 585). It’s commonly used for 1) its comprehensive representation of physical processes, including the carbon cycle and interactive chemistry, provides a robust framework for climate projections, 2) its high performance in the Mediterranean in the Mediterranean region, capturing key climatic features and variability (Eyring et al., 2016), 3) its consistency with CMIP6 and other global climate studies, facilitating comparative analysis and integration with broader research efforts, and 4) its adaptability to simulate different socioeconomic pathways (SSP126 and SSP585) allowing for a comprehensive assessment of future climate impacts under varying conditions (Hourdin et al., 2020; Boucher et al., 2020).

Finally, we applied multicollinearity analysis to avoid model overfitting using ‘usdm’ package (Naimi, 2015) in R 4.3.1 to apply the variance inflation factor (VIF) to exclude the correlated variables with VIF > 5 and a correlation threshold of 0.75 (Guisan et al., 2017). To calculate the relative variables’ importance, we used Pearson’s correlation coefficient in the same package.

### Ensemble modeling and potential habitat suitability

By using the codes [v1 <- vifcor(env, th=0.75 and v2 <- vifstep(env, th=5)], we used the six bioclimatic variables resulted from the multicollinearity test along with the distribution data of the species in R 3.4.1 using ‘sdm’ package (Naimi and Araújo, 2016) to apply ensemble modeling of five common species distribution models (SDMs): generalized linear model (GLM), Random Forest (RF), Boosted Regression Trees (BRT), Support Vector Machines (SVM), and Generalized Additive Model (GAM) which are characterized by high stability compared to other models (Thuiller et al., 2019). In fact, we have applied multiple models and finally picked the ones having AUC value > 0.85 to build an ensemble model through a weighted mean approach (Marmion et al., 2009). We used 70% of the data as training and 30% as testing (Thuiller et al., 2019). We used the recommended Maximum Training Sensitivity Plus Specificity (MTSS) criterion (Liu et al., 2016). To assess model accuracy, we used the area under the curve (AUC) and TSS (Guisan et al., 2017). To estimate the habitat changes between future and current potential predictions, we converted the continuous output maps of current and future habitat suitability into binary maps (presence/absence) depending on the MTSS threshold, and then we applied the equation (Future prediction*2) - (current prediction) to emphasize changes in suitability. This approach highlights areas where habitat suitability increases, decreases, or remains unchanged, providing a clear visualization of habitat dynamics over time.

Finally, this resulted in output, which was visualized as loss, stable, and gain areas in ArcGIS Pro (Dakhil et al., 2021). Model outputs were visualized on ArcGIS Pro (ESRI, USA).

## Results

### Modelling evaluation

**Figure 3:**
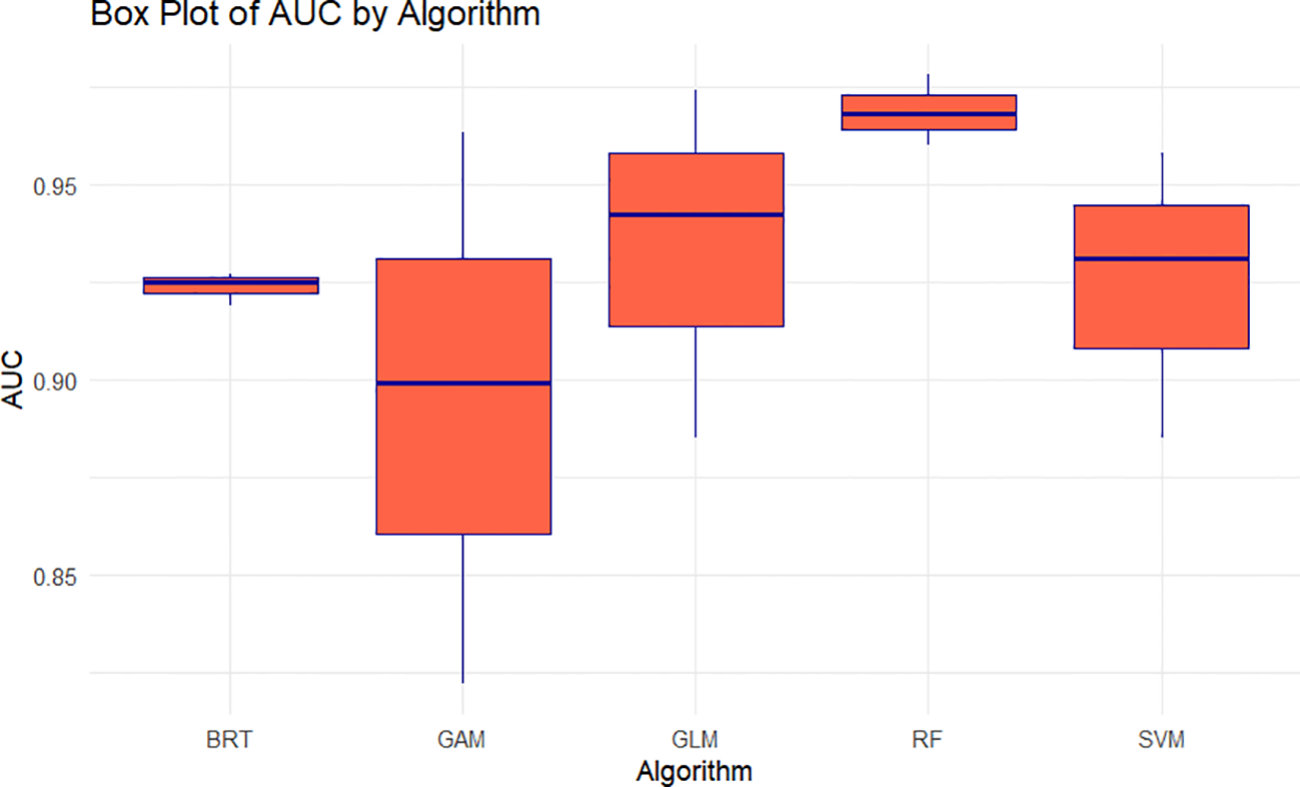
Box Plot of the AUC values obtained from three cross-validation runs on test data for each of the five SDM algorithms used to predict the global distribution of *C. cornigera*.

Ensemble models of BRT, GLM, RF, SVM, and GAM demonstrated exceptional accuracy and performance, with average AUC and TSS of 0.93 and 0.83 respectively. The SVM and GLM algorithms performed better than the other algorithms. In the species studied, the ensemble models had the best overall performance with mean TSS (= 0.85), while RF and GLM algorithms performed better than the other algorithm with mean AUC (≥ 0.93) (**Table 1**; **Figure 3**).

**Table 1:** Performance of the model algorithms.

Methods	AUC	COR	TSS	Deviance
brt	0.92	0.65	0.84	0.22
glm	0.93	0.61	0.85	0.25
rf	0.97	0.77	0.84	0.14
svm	0.92	0.81	0.85	0.15
gam	0.89	0.69	0.78	1.48

**Figure 4:**
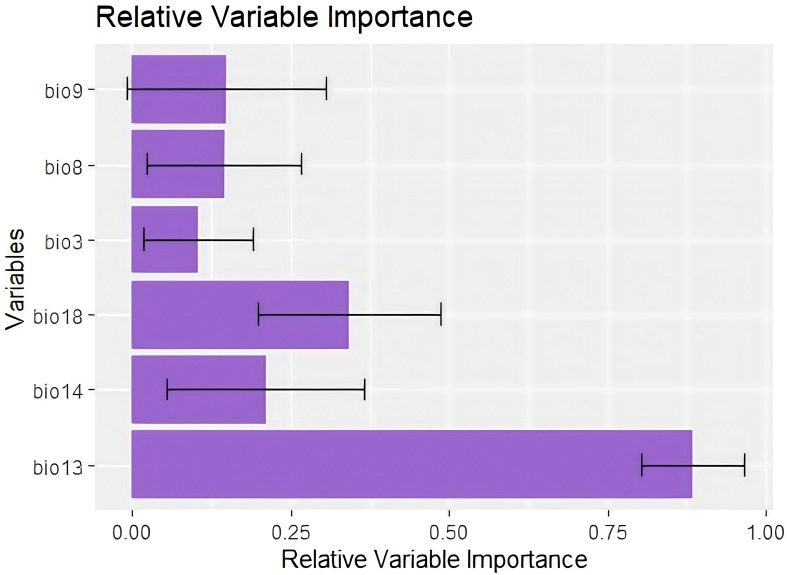
Relative variable importance of the selected variables. The whiskers represent the range of the data outside the interquartile range (IQR).

**Figure 5:**
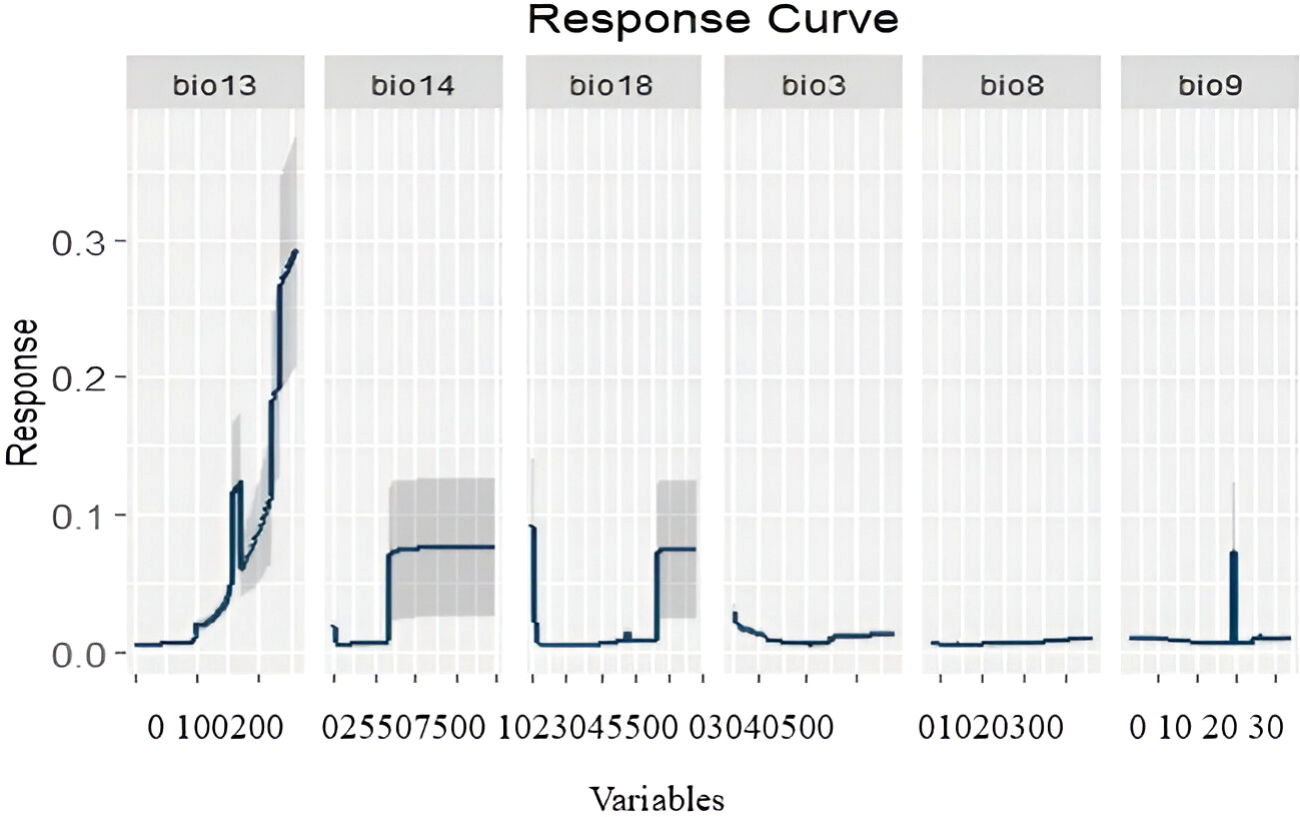
Response curves of the predictor variables used in the distribution modelling of *C. cornigera*.

Multicollinearity analysis of the total 19 predictors resulted in six uncorrelated variables with VIF > 5 and a correlation threshold of 0.75 (**Table 2**), which had been used in the ensemble modeling. According to Pearson’s correlation coefficient, Precipitation of the wettest month (Bio13) (88.3%), Precipitation of the warmest quarter (Bio18) (30%), and Precipitation of the driest month (Bio14) (22%) were the most important variables explaining the potential distribution of *C. cornigera* with Relative Importance higher than 21% up to 88.3% (**Figure 4**). The response curves revealed that with the increase of the Precipitation of the wettest month (Bio13), the probability of presence increases (**Figure 5**). In contrast, the probability of presence decreases by Isothermality (Bio2/Bio7) × 100 (Bio3) (-0.103 correlation value indicating a weak negative correlation).

**Table 2:** Model summary of the selected climate predictor variables explaining the potential distribution of *C. cornigera* in the Mediterranean region.

Code	Variable	VIF
Bio13	Precipitation of wettest month	2.5
Bio14	Precipitation of Driest Month	3.2
Bio18	Precipitation of Warmest Quarter	2.3
Bio3	Isothermality	2.1
Bio8	Mean Temperature of Wettest Quarter	3.3
Bio9	Mean Temperature of Driest Quarter	1.5

To prevent multicollinearity issues, related variables with variance inflation factor (VIF) values greater than five and a correlation threshold of 0.75 were eliminated.

**Figure 6:**
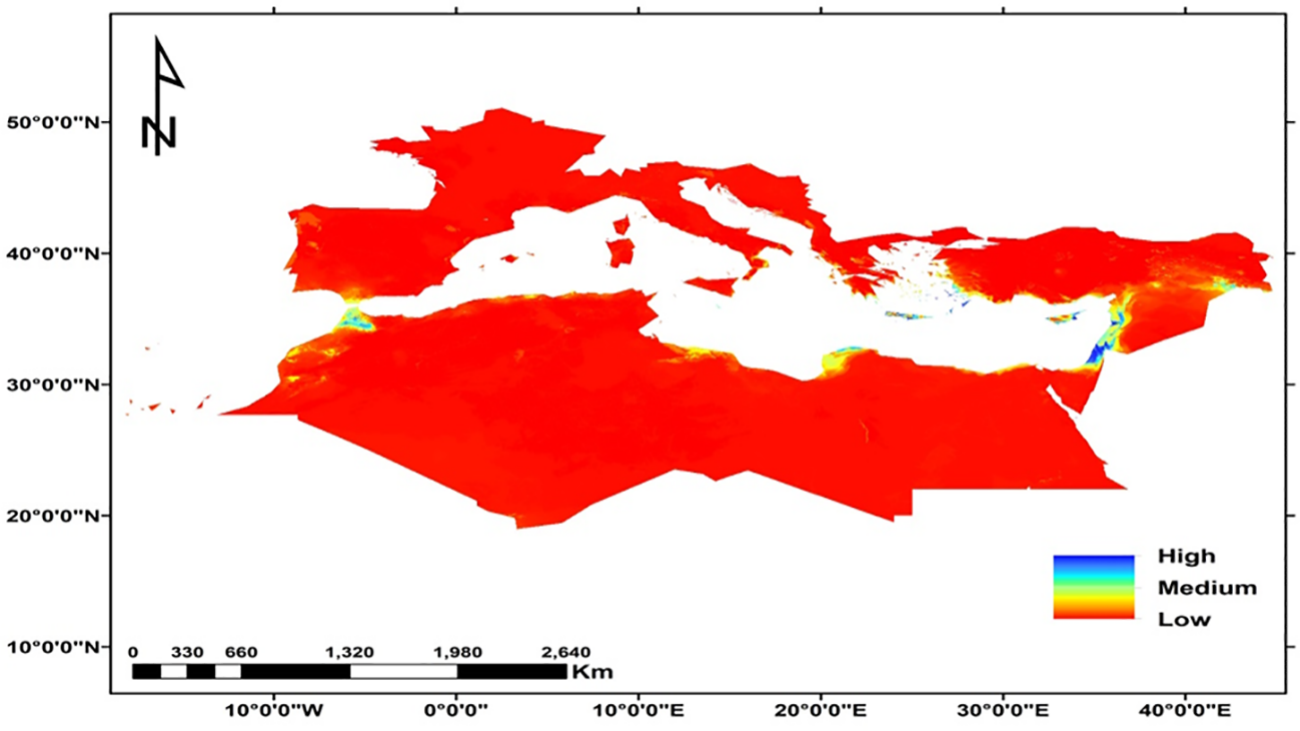
The habitat suitability map of *C. cornigera* under the current climate conditions at the global scale.

Ensemble habitat suitability map illustrating the area of the currently suitable habitats for *C. cornigera* based on the MTSS threshold 0.076 was 10298 km^2^. It showed high habitat suitability in Egypt, (especially the Arishian sub sector), Palestine, Morocco, Northern Cyprus, and different islands in the Sea of Crete (**Figure 6**).

**Figure 7:**
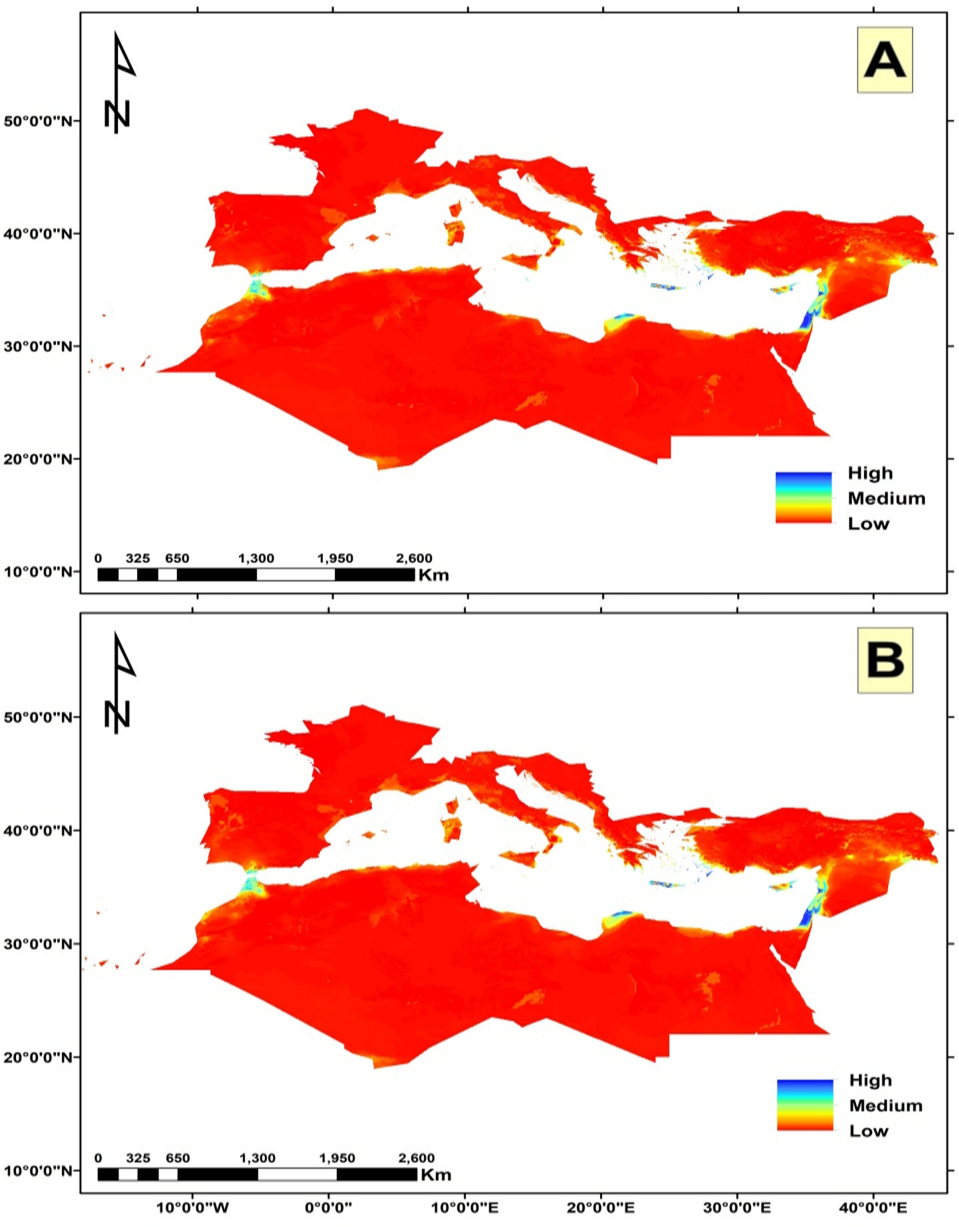
The habitat suitability map of *Cynara cornigera* under the two different climate change scenarios. **(A)** represents the SSP126 scenario; and **(B)** the SSP585 scenario projected for 2021-2040 period.

**Figure 8:**
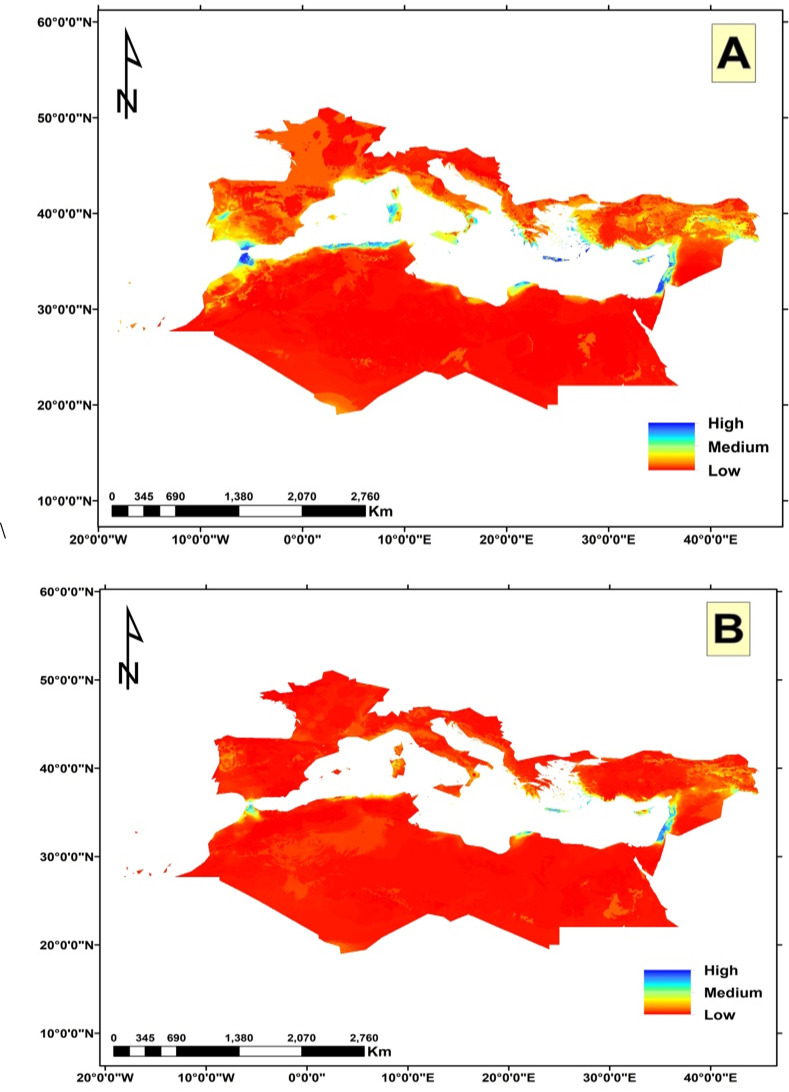
The habitat suitability map of *Cynara cornigera* under the two different climate change scenarios. **(A)** represents the SSP126 scenario; and **(B)** the SSP585 scenario projected for 2061-2080 period.

The potential future changes in habitat suitability were slightly similar under both climate change scenarios (**Figures 7**, **8**). Both showed a loss in the potential distribution of *C. cornigera*. By 2030, the model predicts a reduction of suitable habitat by 34 km^2^ under SSP 126 scenario, while only 30 km^2^ will be lost under SSP585. Whereas the gain areas were very neglectable under both scenarios (only 4 km^2^). Nevertheless, 10267 km^2^ would remain suitable for this species, which is approximately 28 percent of the total study area. It showed high habitat suitability in Libya, Egypt, (especially the Arishian sub sector), Palestine, Northern Cyprus, and different islands in the Sea of Crete. On the other hand, the future potential distribution area of *Cynara cornigera* (SSP 126 scenario) for 2070 showed that there is an increase in the suitable habitats area and habitat suitability distribution along the Mediterranean coastal strip of Spain, Sardinia, Morocco, Algeria, Tunisia, Libya, Egypt, (especially the Arishian sub sector), Palestine, Lebanon, Northern Cyprus, and different islands in the Sea of Crete. On the other hand, the future potential distribution in SSP 585 scenario showed decrease in the suitable habitats area and habitat suitability distribution that is restricted to small sector of the Mediterranean coastal strip of Libya, Egypt, Palestine and Lebanon, while it has medium habitat suitability in Morocco, Northern Cyprus, and different islands in the Sea of Crete.

**Figure 9:**
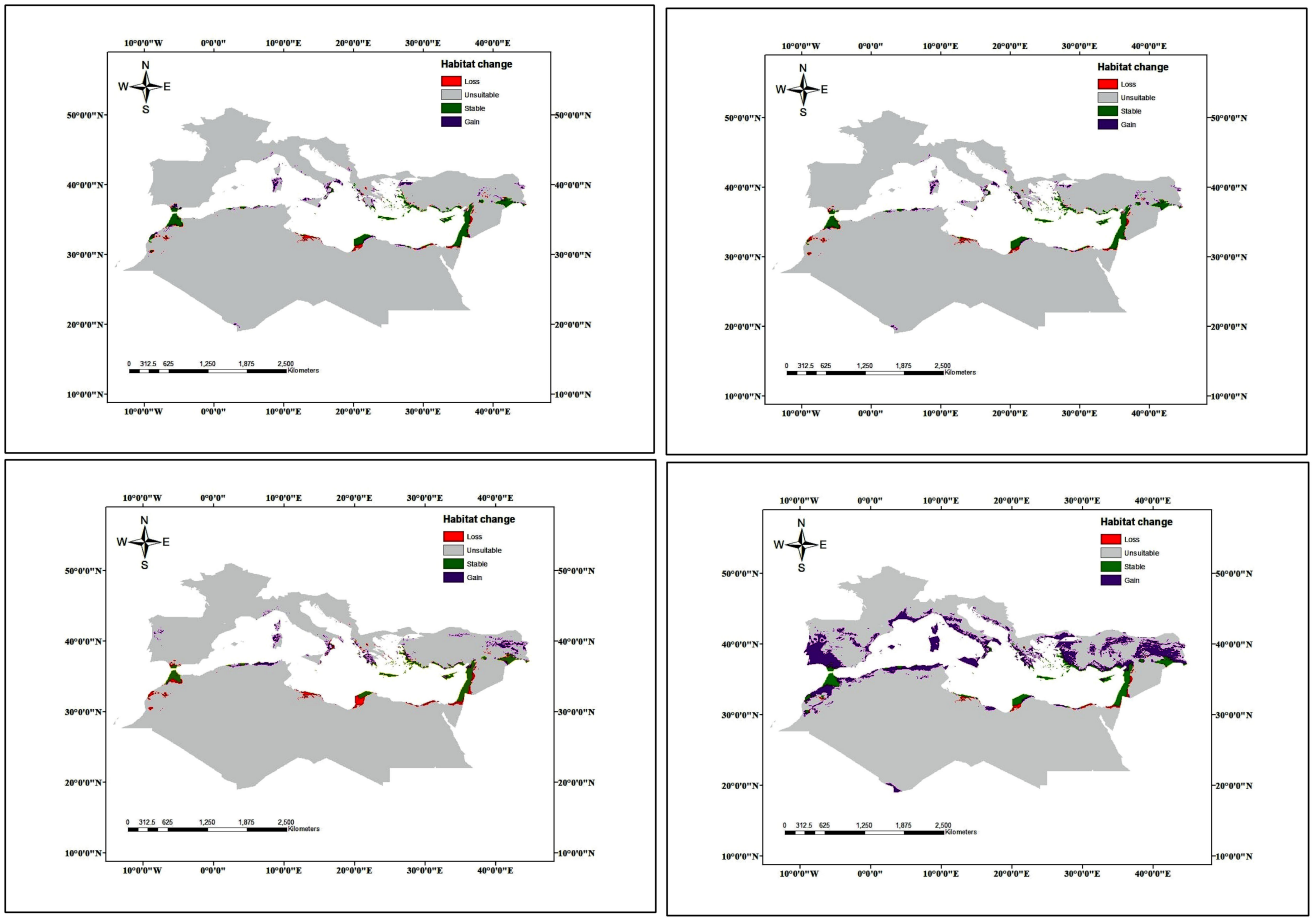
Possible habitat modifications under the two scenarios of climate change **(A)** SSP126_2021-2040, **(B)** SSP585_2021-2040, **(C)** SSP126_2061-2080, and **(D)** SSP585_2061-2080.

The potential changes in habitat suitability were slightly similar under both near future climate change scenarios (SSP126_2021-2040 and SSP585_2021-2040). Both showed a loss in the potential distribution of *C. cornigera* with a percentage of 19.8% and 21.1% respectively. The gain areas in the future were small (21.6% and 22.6%) northern Algeria and southern Europe (**Figures 9A, B**). The climatically stable areas (green colors) were found in Palestine, Marmarica and Gibraltar (**Figures 9A–D**). On the other hand, under the far future low scenario of climate change (SSP126_2061-2080), the loss area was lower (14.9%) compared to the near future scenarios. Indeed, there was a noticeable increase in gain areas under the scenario of SSP126_2061-2080 to 78.4%. In the meantime, there was a dramatic increase in loss areas (34.2%) and decrease in gain areas (30.2%) under the highest far future scenario of SSP585_2061-2080.

## Discussion

Climate change has been shown to have a significant impact on local extirpations, colonization, and shifts in the distribution of plant species (Parmesan and Hanley, 2015). So, this study uses species distribution models (SDMs) to forecast potential changes in species distributions due to climatic change.

*C cornigera*, a species within the Cynara genus, exhibits limited dispersal ability primarily due to its indehiscent dry fruits. These fruits are not adapted for long-distance dispersal mechanisms such as wind or water transport (thalassochory). As a result, the species relies heavily on local dispersal processes, which can restrict its ability to colonize new areas. This limited dispersal capacity is compounded by the specific habitat requirements of *C. cornigera*, which further confines its distribution to suitable microhabitats within its native range. Understanding these dispersal limitations is crucial for predicting the species’ potential distribution under changing climatic conditions and for developing effective conservation strategies. Cuena-Lombraña et al. (2024) highlight that indehiscent dry fruits, like those of *C. cornigera*, have limited dispersal ability via thalassochory, which should be considered when predicting the species’ potential distribution. Integrating these factors will enhance the robustness of species distribution models and provide a more comprehensive understanding of the species’ ecological dynamics. Indeed, Cuena-Lombraña et al. (2024) suggest that propagules of coastal plant species do not seem to have a consistently higher buoyancy and seed viability after saltwater immersion than inland species. However, they observed differences between widely distributed and endemic plants, suggesting that their current distribution is likely correlated with their long-distance dispersal ability. The estimated long-distance dispersal provides insights into the potent. This maybe explains the possible presence of vicariant species in areas, such as those in the western Mediterranean, where their models have found suitable conditions. In fact, the majority of Cardueae diversification episodes are associated with the ongoing cycles of area connection and division between the western Mediterranean Basin and the Anatolian microplate during the Oligocene-Miocene, as well as the uplift of the Himalayan range starting in the Miocene. Most likely during the colder Pliocene-Pleistocene period, thistles spread and colonized the remaining continents (such as the New World, Africa, and Australia) from these two locations (Barres et al., 2013).

Our findings indicate that the models utilized in this study are effective in identifying suitable habitats for *C. cornigera*’s global distribution. Species distribution models are used by many studies such as Ehrlén and Morris (2015); Srivastava et al. (2019); Di Sora et al. (2023) to find appropriate areas for the presence of plant species. Because there is no perfect approach for species distribution modelling, we employed the ensemble approach that include various algorithms. Ensemble models demonstrated strong prediction performance based on AUC and TSS in this study. This result agrees with Marmion et al. (2009) who stated that it is expected that applying the ensemble approach to combining and average models will raise model certainty and improve model robustness when applied to properly modelling species distributions. Bedair et al. (2023) used ensemble species distribution models with high accuracy for mapping the habitat suitability and weighted endemism of Mediterranean endemics in the Egyptian Mareotis subsector and estimation of the environmental factors affecting the distribution of these taxa. Moreover, Khan and Verma (2022) utilized Ensemble SDMs to predict the impact of future climate change on the global distribution of *Olea europaea* subsp. *cuspidata* and found that ensemble modelling could be immensely useful in identifying hotspots of cultivation for the successful rehabilitation and preservation of this olive lineage under future climate scenarios. However, Hao et al. (2020) observed no significant benefit in employing ensembles over individual algorithms. Our results predicted that the Precipitation of the wettest month (Bio 13), the Precipitation of the warmest quarter (Bio 18), and the Precipitation of the driest month (Bio 14) could potentially be considered as constraints on the possible geographic range of *C. cornigera*. In fact, Precipitation of the Wettest Month (Bio 13) captures the maximum monthly precipitation, which is critical for plant species that rely on peak water availability for growth and reproduction. For *C. cornigera*, which thrives in Mediterranean climates characterized by wet winters and dry summers, the wettest month’s precipitation ensures sufficient water supply during the critical growth period (Noce et al., 2020). In addition, Precipitation of the Warmest Quarter (Bio 18) measures the total precipitation during the warmest three months of the year. In the Mediterranean region, summer months can be extremely dry. The availability of water during this period is essential for the survival and resilience of *C. cornigera*, helping it withstand drought conditions and maintain physiological functions (Noce et al., 2020). Further, Precipitation of the Driest Month (Bio 14) indicates the minimum monthly precipitation, highlighting the extreme dry conditions that the species must endure. Understanding this helps in assessing the drought tolerance of *C. cornigera* and its ability to survive in the harshest part of the year (Noce et al., 2020). In fact, in studies on the distribution of various plant species in the Mediterranean, it was found that bioclimatic variables related to precipitation were among the most important predictors. This includes variables like Bio 13, Bio 14, and Bio 18, which help in understanding the ecological niches and potential distribution areas under current and future climate scenarios (Rego and Rocha, 2014). Furthermore, a study on *Quercus ilex* (Holm oak), has shown that precipitation variables, particularly those related to seasonal extremes, are significant predictors of species distribution. For instance, research has demonstrated that the distribution of *Q. ilex* is strongly influenced by precipitation during the driest and wettest months (Yilmaz et al., 2024).

Recent studies have shown how rainfall has a major role in determining the geographic distribution of many plant species such as *Mentha pulegium* L. in Tunisia (Soilhi et al., 2022), *Platanus orientalis* L. in Turkey (Kamer Aksoy, 2022a) and *Juniperus phoenicea* in Mediterranean and Macaronesian regions (Salvà-Catarineu et al., 2021). These results can be reinforced by the fact that an area’s climate has a crucial role in population growth (Hansen et al., 2018).

Some other studies elucidated that some factors could affect the distribution of plant species in addition to the bioclimatic variables. In Spain and Morocco, Masso et al. (2018) found that the most important climatic variables to predict the niche of *C. baetica* subsp. *baetica* were pH and precipitation, while for *C. baetica* subsp. *maroccana*, the variables were precipitation and elevation. In fact, *C. cornigera* shows a marked preference for saline depressions and low elevation plains due to several ecological and physiological factors. These areas provide saline soils, which *C. cornigera* is well-adapted to, allowing it to thrive where other species might struggle. The depressions and plains also collect water, ensuring a more consistent moisture supply crucial for growth in the variable Mediterranean climate. Additionally, these low-lying areas offer more stable temperatures and reduced wind exposure, which help minimize water loss through evaporation and transpiration. By occupying these specific habitats, *C. cornigera* can avoid competition with less salt-tolerant species, effectively carving out a unique ecological niche. Similar patterns are observed in other Mediterranean endemics, such as species of the genus Limonium, which also favor saline environments. These factors collectively make saline depressions and low elevation plains ideal habitats for *C. cornigera*, supporting its survival and proliferation in the Mediterranean region (Bedair et al., 2023a). The key variables affecting the global potential distribution of *Juniperus phoenicea* are aridity, temperature, seasonality, and clay content according to Dakhil et al. (2022). The findings of Aouinti et al. (2022) demonstrated that the main factors responsible for the current distribution the Montpellier Maple in the Mediterranean and Middle Western Asian regions were temperature seasonality, elevation, mean annual temperature, mean annual precipitation, and maximum temperature of the warmest month. Human activities have significantly affected the global dispersion of alien plant species according to (Wang and Xu, 2016). Moreover, topography and geomorphology are found to be controlling factors of *Euphorbia polygalifolia*, *Festuca eskia*, *Genista obtusiramea*, *Juncus trifidus*, *Luzula caespitosa* and *Vaccinium uliginosum* in Northern Spain (Bedia et al., 2011). According to Kamer Aksoy (2022b), environmental management techniques, hydrological characteristics, climatic conditions and external variables like human intervention all affect the distribution of the carob (*Ceratonia Siliqua* L.) in Turkey. Furthermore, a study on *Quercus ilex* (Holm oak), has shown that precipitation variables, particularly those related to seasonal extremes, are significant predictors of species distribution. For instance, research has demonstrated that the distribution of *Q. ilex* is strongly influenced by precipitation during the driest and wettest months (Yilmaz et al., 2024). Our study’s models indicated that the current suitable habitats for *C. cornigera* are found in Palestine, Morocco, Egypt (particularly in the Arishian sub sector), Northern Cyprus, and various islands in the Sea of Crete. Tzanoudakis and Kypriotakis (1987); Della et al. (2006); Hegazy et al. (2015a, b), El Sohafy et al. (2016); El hasham et al. (2020); Shamso et al. (2021); Sumengen Ozdenefe et al. (2022) and Bedair et al. (2024a, b) reported that this species is found in the same habitats predicted by our models in this study. Under the two scenarios SSP126 and SSP585 For the period of 2021-2040, the suitable habitats of this species will decrease, and the high habitat suitability will be found in Libya, Egypt, (especially the Arishian sub sector), Palestine, Northern Cyprus, and different islands in the Sea of Crete. This finding agrees with Soilhi et al. (2022) who reported that the moderately and highly appropriate habitats for *Mentha pulegium* in Tunisia will decline in the 2050s and 2070s under the two RCPs (RCP2.6 and RCP8.5). Khan et al. (2022) also found that suitable areas of *Pinus gerardiana* will decrease by about 94% between 2060 and 2080 under the RCP 8.5 scenario in south Asia. The decline in the appropriate habitat could be attributed to the gradual decrease in precipitation, that directly impacts availability of water for plants (Shi et al., 2021). Moreover, Precipitation impacts the longitude distribution of species, and the high temperature mostly affects their latitude distribution (Zhang H. et al., 2020). The recent episodes of anomalous drought and heath waves will cause the fragmentation of the suitable habitats for this species (Matusik et al., 2013). On the other hand, the future potential distribution area of *C. cornigera* (SSP 126 scenario) for 2061 and 2080 showed that there is an increase in the suitable habitats area and habitat suitability distribution. Beca-Carretero et al. (2020) found that climate change is expected to significantly expand the biogeographical range of *Halophila stipulacea* and *Halophila decipiens*, with 80% of the Mediterranean coastline expected to be suitable for colonization based on salinity conditions and temperature alone. The expansion of suitable habitat may occur because the species often moves to high-altitude habitats. Increased precipitation and global warming may make high-altitude locations more suited to the growth of the species.

Variation in the timing of rain events during the rainy season frequently leads to mid-season droughts with varying durations (Mulroy and Rundel, 1977). According to current predictions, the Mediterranean region will face an increase in temperature and drought (IPCC, 2007). The plant soil system could face changes in feedback and equilibrium if there is a severe drought combined with irregular heavy rainfall (Frei et al., 1998). Many crop species, like artichokes, are particularly vulnerable to drought stress because of their high transpiration rate, which inhibits photosynthesis and other metabolic processes that impact crop quality and production (Allahdadi and Bahreininejad, 2019). In fact, climate and vegetation data indicate that the border between the Mediterranean and temperate belts has shifted from 1980 to 2020 upward by 150 and 300 meters, respectively. The frequency of dry-adapted species is increasing at low elevations, while the moisture requirements of vegetation are increasing at high elevations. Responses are quicker at low elevations, according to a comparison of climatic data and vegetation responses (Saatkamp et al., 2023).

Finally, based on our results, several recommendations can be made for *C. cornigera’s* management and conservation. The predicted reduction in suitable habitat by 2030 under both SSP126 and SSP585 scenarios highlights the need for proactive conservation strategies. It is crucial to prioritize the protection of the remaining 10,267 km² of suitable habitat, which constitutes approximately 28% of the total study area. Conservation efforts should focus on regions with high habitat suitability, such as Libya, Egypt (especially the Arishian sub-sector), Palestine, Northern Cyprus, and various islands in the Sea of Crete. Additionally, habitat restoration and management practices should be implemented to mitigate the loss of suitable areas and enhance the resilience of existing populations. Monitoring and adaptive management will be essential to respond to the dynamic changes in habitat suitability, particularly under the more severe SSP585 scenario, which predicts a significant decrease in suitable habitats by 2080. Collaborative efforts across the Mediterranean region, involving local communities, governments, and conservation organizations, will be vital to ensure the long-term survival of *C. cornigera* in the face of climate change. The output maps from this study can be used by the administrative authorities to determine the areas of high risk and organize conservation efforts there. They can also utilize this information to create management and conservation plans, as well as to survey and monitor their efforts in the future.

## Conclusion

The study explored the current distribution and predicted suitability of *C. cornigera* across the Mediterranean region’s diverse ecosystems under various climate change scenarios using SDMs. The study revealed how climate change may influence *C. cornigera*, identifying regions at risk of species range shifts and loss. Results demonstrated the intricate relationships between habitat suitability and environmental variables, highlighting the importance of factors like temperature and precipitation. Our research specifically showed that the precipitation of the wettest month, the precipitation of the warmest quarter, and the precipitation of the driest month were the most parameters that impacted the distribution of *C. cornigera* throughout the Mediterranean basin. The importance of these varied habitats was highlighted by the identification of habitat suitability hotspots in Palestine, Morocco, Northern Cyprus, Egypt (particularly the Arishian sub sector), and various islands in the Sea of Crete. Notably, the decline in habitat suitability under future climate scenarios signifies the urgent need for conservation efforts, especially considering the threats posed by human activities such as land use change and habitat fragmentation. These findings underscore the importance of targeted conservation efforts in climatically stable areas like Palestine, Marmarica, and Gibraltar, and adaptive strategies to mitigate habitat loss under more severe climate change scenarios. This comprehensive analysis provides a crucial foundation for future conservation planning and ecological studies of *C. cornigera*. The study’s findings underscore the importance of adaptive conservation planning to safeguard Mediterranean endemic species, particularly endangered species identified through early surveys and assessments. These strategies should place a high priority on habitat protection, restoration, sustainable land management techniques, and efforts to mitigate and adapt to climate change. Furthermore, the prediction of increased future suitable conditions is also interesting, probably because this species prefers dry conditions, which are perhaps expanding throughout the Mediterranean. In general, the amalgamation of SDMs with empirical data yields valuable insights for guiding conservation strategies in the context of ongoing climate change and human disturbances in ecologically comparable ecosystems.

## Data Availability

The original contributions presented in the study are included in the article/supplementary material. Further inquiries can be directed to the corresponding author/s.
